# Production of ultracold polyatomic molecules with strong polarity by laser cooling: A detailed theoretical study on CaNC and SrNC

**DOI:** 10.3389/fchem.2022.1009986

**Published:** 2022-09-23

**Authors:** Wensha Xia, Jianwei Cao, Qing Lu, Wensheng Bian

**Affiliations:** ^1^ Beijing National Laboratory for Molecular Sciences, Institute of Chemistry, Chinese Academy of Sciences, Beijing, China; ^2^ School of Chemical Sciences, University of Chinese Academy of Sciences, Beijing, China

**Keywords:** molecular laser cooling, ultracold polyatomic molecules, large polarity, ab initio, Franck-Condon factor

## Abstract

Laser cooling molecules to the ultracold regime is the prerequisite for many novel science and technologies. It is desirable to take advantage of theoretical approaches to explore polyatomic molecular candidates, which are capable of being cooled to the ultracold regime. In this work, we explore two polyatomic candidates, CaNC and SrNC, which are suitable for laser cooling. These molecules possess impressively large permanent dipole moments (∼6 Debye), which is preferred for applications using an external electric field. High-level ab initio calculations are carried out to reveal electronic structures of these molecules, and the calculated spectroscopic constants agree very well with the available experimental data. For each molecule, the Franck-Condon factor matrix is calculated and shows a diagonal distribution. The radiative lifetimes for CaNC and SrNC are estimated to be 15.5 and 15.8 ns, respectively. Based upon the features of various electronic states and by choosing suitable spin-orbit states, we construct two feasible laser cooling schemes for the two molecules, each of which allows scattering nearly 10000 photons for direct laser cooling. These indicate that CaNC and SrNC are excellent ultracold polyatomic candidates with strong polarity.

## 1 Introduction

Cooling molecules to the ultracold regime is of significant importance for novel science and techniques. Representative applications include precision measurements, quantum information storage, and quantum computing (Tarbutt, 2018; Isaev, 2020). For a long time, however, laser cooling to ultracold regimes has been limited to atoms and diatomic molecules. This obstructs further development of those next-generation techniques. So, it is desirable to extend laser cooling studies to polyatomic molecules. On the adventure of exploring polyatomic molecular candidates for direct laser cooling, the Doyle group and the Berger group suggest several polyatomic molecules suitable for direct Doppler cooling (Isaev and Berger, 2016; Kozyryev et al., 2016). Then, Doyle and co-workers contribute a series of milestone work such as experimentally realizing laser cooling SrOH and CaOCH_3_ to the ultracold regime (Kozyryev et al., 2017; Baum et al., 2020; Mitra et al., 2020). Further studies have also been carried out such as cooling CaOH to the sub-Doppler temperature, accurate determination of vibrational branching ratios (VBRs) and radiative lifetimes for YbOH (Baum et al., 2021; Vilas N et al., 2022). Other theoretical and experimental groups have also made important contributions to this emerging field (O'Rourke and Hutzler, 2019; Isaev et al., 2017; Liu et al., 2021; Xia et al., 2021; Paul et al., 2019; Ivanov et al., 2020a; Ivanov et al., 2020b; Ivanov et al., 2020c).

It is clear that much still awaits to be explored in searching for polyatomic candidates which can be cooled to the ultracold regime. Molecular candidates for laser cooling should meet criteria proposed by Di Rosa, although the criteria were originally brought up for diatomic molecules (Di Rosa, 2004). Based on the three criteria proposed by Di Rosa, candidate molecules should have a highly diagonal Franck-Condon factor (FCF) matrix or vibrational branching ratios. In addition, the candidate molecule should have a short lifetime for the upper state. Thirdly, it is required that there exists no interference from the intermediate electronic state. Recently, Bian proposed the fourth criterion that there should be no electronic-state crossing nearby the states of interests (Li et al., 2020).

Here, accurate *ab initio* calculations are carried out for the triatomic molecules, CaNC and SrNC. The results show that CaNC and SrNC meet the four criteria mentioned above, which reveals that they are excellent ultracold polyatomic candidates. We have constructed feasible laser cooling schemes for them, which are able to bring them to the ultracold regime. As will be shown later, these molecules possess impressively large permanent dipole moments (PDM); for instance, the dipole moment of CaNC is more than 6 Debye. To the best of our knowledge, these dipole moments are larger than those of other known ultracold candidates for laser cooling. Such a strong molecular polarity is very useful, particularly in applications applying external electric fields (Isaev et al., 2017). It is worth adding that the CaSH molecule also has large dipole moments (∼5.5 Debye) which has been proposed for direct laser cooling (Augenbraun et al., 2020), although it has a bent configuration.

The previous studies on CaNC and SrNC mainly focus on their spectroscopic properties. For the CaNC molecule, the adiabatic excitation energy has been determined by the laser-induced fluorescence spectroscopy (Douay and Bernath, 1990; Whitham et al., 1990; Steimle et al., 1992; Scurlock et al., 1994) as well as theoretical calculations (Bauschlicher et al., 1985; Nanbu et al., 1997; Ishii et al., 2003; Isaev and Berger, 2016). The vibrational spectroscopic constants of CaNC have been experimentally measured by Andrews and co-workers (Lanzisera and Andrews, 1997). Its vibrational frequencies were also calculated at different levels of theories and basis sets (Lanzisera and Andrews, 1997; Nanbu et al., 1997; Ishii et al., 2003; Isaev and Berger, 2016). In addition, high-lying excited states have been studied by the ultraviolet laser spectroscopy (Greetham and Ellis, 2000a). For the SrNC molecule, less has been explored. Its A^2^Π→X^2^Σ^+^ transition has been studied by the laser spectroscopy (Douay and Bernath, 1990), and the vibrational frequencies for the ground state were experimentally determined by the infrared spectroscopy (Lanzisera and Andrews, 1997). Its high-lying excited states have been studied by the jet-cooled laser-induced fluorescence spectroscopy (Greetham and Ellis, 2000a; Greetham and Ellis, 2000b), although the geometry and spectroscopic information for its first excited state is still unavailable.

So far, the laser cooling studies on CaNC and SrNC have been very limited. Isaev *et al.* (Isaev and Berger, 2016) carried out an interesting study involving the calculation of FCFs for CaNC, however, the laser cooling scheme was not constructed in that work and a further detailed study is required. In particular, it is important to examine if there are nearby electronic states intervening the first excited state, and to take the spin-orbit coupling effect into account. On the other hand, no laser cooling studies have been carried out for SrNC.

In this work, we carry out detailed theoretical investigations on laser cooling of CaNC and SrNC. The article is organized as follows: The computational details are described in the “Methods and computational details” section. In the “Results and Discussion” section, the potential energy curves (PECs) of CaNC and SrNC are first described. Next, the spectroscopic as well as other molecular properties are summarized and compared to experimental values. Lastly, the cooling schemes are constructed for the two molecules. Conclusions are given at the end of the article.

## 2 Methods and computational detail

**FIGURE 1:**
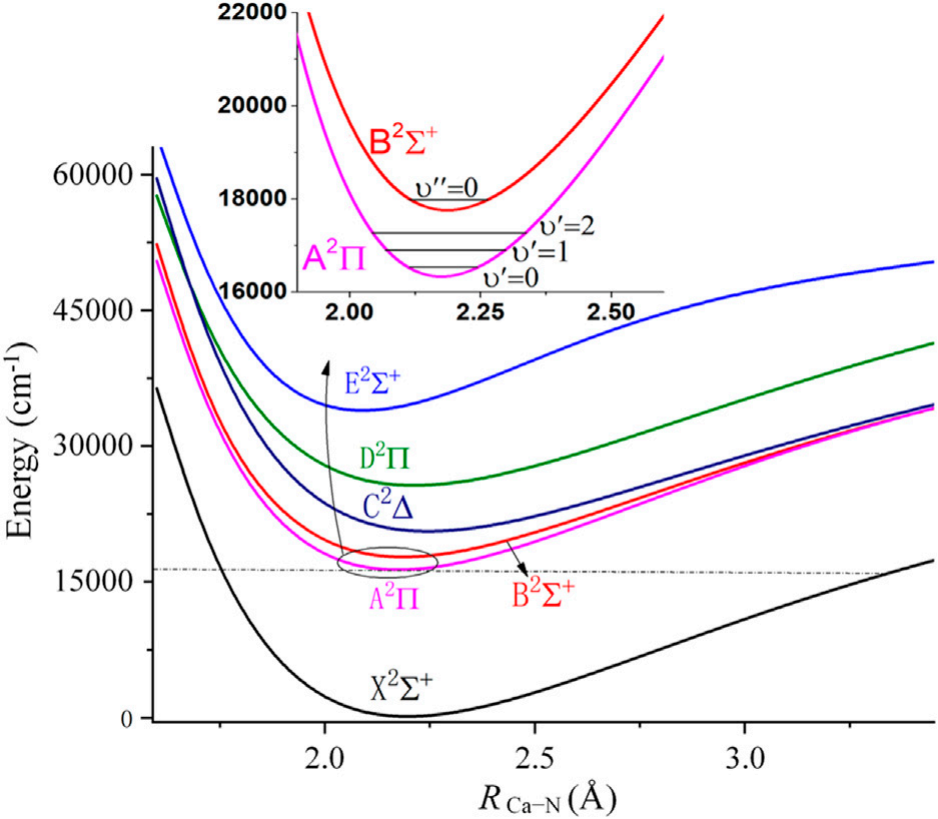
Potential energy curves of linear CaNC along R_Ca-N_, with R_N-C_ fixed at 1.183Å. The gray dashed line shows that the minimum of the A state is well below the dissociation limit of the X state.

In this work, the potential energy curves are constructed based on ∼35 grid points for both CaNC and SrNC. To calculate the energy of each grid point, the constrained optimization is carried out by scanning the M-N distance while freezing the N-C bond length as 1.183 Å and bond angle as 180°. A relaxed scanning of the M-N (M = Ca or Sr) distance is also performed while optimizing the N-C bond length and the bond angle. The electronic energies are calculated by the internally contracted multi-reference configuration interaction method with the Davidson correction (icMRCI + Q) running after the state-averaged complete active space self-consistent field method (SA-CASSCF) (Werner and Knowles, 1988; Knowles and Werner, 1992). The C_2v_ symmetry is used for the constrained PEC scanning, while the C_s_ symmetry is used for the un-constrained PEC scanning. The selection of active space is very important for the icMRCI + Q calculations (Yu and Bian, 2012; Feller et al., 2014; El-Kork et al., 2017; Zeid et al., 2020; Li and Bian, 2021; Moussa et al., 2021). Here, the chosen active space includes 7 electrons in 13 orbitals for CaNC or SrNC. The active space includes four a_1_, four b_1_, four b_2_, and one a_2_ molecular orbitals in the C_2v_ symmetry, which involves the 4s electron on Ca (or 5s electron on Sr) and the π and “lone-pair” electrons on NC for the linear M-NC system (M = Ca or Sr). For the PEC calculations as shown in Figure 1, in total 9 states with equal weights are used for the state-averaged calculations. For the frequency calculations, the lowest 4 states are included for the state-averaged calculation with equal weights. The size of basis sets is also important in improving the accuracy of calculations (Feller et al., 2008; Liu and Bian, 2008; Liu et al., 2009; Yu and Bian, 2011). In this work, the def2-QZVP basis set is used for Ca and the dhf-QZVP basis set is used for Sr (Weigend et al., 2003; Weigend and Baldes, 2010), while the aug-cc-pv5z basis sets are used for N and C (Dunning, 1989; Kendall et al., 1992). The spin-orbit coupling effect is calculated by using the Breit-Pauli operator with the state-interacting method (Berning et al., 2000). The PDM are calculated at the same level of theory and basis sets as PEC calculations.

The local potential energy surfaces around the equilibrium geometries are constructed by fitting ∼50 grid points. Each local potential energy surface is fitted by a 5^th^ order polynomial in the internal displacement coordinate (Lu, 2020; Lu and Peterson, 2020). The energy of each grid point is calculated by the icMRCI + Q method. The active space and basis sets are kept the same as the PEC calculations. The optimized geometries and calculated frequencies are similar using different methods.

The FCFs are calculated as the square of the overlap of electronic and vibrational wavefunctions. The Born-Oppenheimer (BO) approximation is accordingly used. Although explicit considerations of the vibronic coupling effect could increase the accuracy for branching ratio calculations, such as those from the study of CaOH (Zhang et al., 2021), the numerical changes due to the breakdown of BO approximation are expected to be very small, since only heavy atoms and the A (000) state are involved in this work (the hydrogen atom is involved in the CaOH study (Zhang et al., 2021)). Due to the selection rules, the rotational branching can be eliminated by driving the *J* = 1 → *J'* = 0 transition for each vibrational level, where *J* is the rotation quantum number for each vibrational level and the primed label refers to the excited electronic state (Fitch et al., 2021). Based on the calculated FCFs, the radiative lifetime (*τ*) for the transition is calculated by Isaev et al. (2017)):

$$\tau =\frac{4.936\times 10^{5}}{{\left|TDM\right|}^{2}\sum_{\nu }{\left(FC\right)}_{\nu \nu^{\prime }}\Delta E_{\nu \nu^{\prime }}^{3}}\frac{g^{\prime }}{g^{\prime \prime }}$$ (1)

where FC is the FCF for each vibronic transition *υυ′*, and the transition dipole moment (TDM) is calculated at the equilibrium geometry of the ground state using the same level of theory and basis sets as the PEC calculations mentioned above. The *∆E*_*υυ'*_ is the energy difference between the vibronic levels *υυ′*. *g′* and *g″* are the degeneracies of upper and lower states, respectively. In addition, the VBRs are calculated following a previous study (Lefebvre-Brion and Field, 2004).

The Doppler temperature (*T*_*Doppler*_) is evaluated by Zeng et al. (2001):

$$T_{Doppler}=\frac{h}{4k_{B}\pi \tau }$$ (2)

where *h* and *k*_*B*_ are the Planck constant and the Boltzmann constant, respectively. And *τ* is the radiative lifetime as calculated above.

The recoil temperature (*T*_*recoil*_) is approximated as (Cohen-Tannoudji, 1998):

$$T_{\mathrm{recoil}}=\frac{h^{2}}{mk_{B}\lambda^{2}}$$ (3)

where *m* is the relative molecular mass, and *λ* is the wavelength for the main transition channel.

The fitting of PECs is carried out by the LEVEL program (Le Roy, 2017). The FCFs are calculated by the ezFCF package (Gozem and Krylov, 2021). All remaining calculations are performed by the Molpro package (Werner et al., 2012; Werner et al., 2015; Werner et al., 2020).

## 3 Results and discussion

### 3.1 Potential energy curves

In order to be laser cooled to the ultracold regime, the molecules are required to have no interference from intermediate electronic states. It is well-known that the isocyanide ligand has a large energy gap between the highest occupied molecular orbital (HOMO) and the lowest occupied molecular orbital (LUMO), while the HOMO-LUMO energy gap for alkaline earth metals is relatively small. Therefore, the interfering states should mainly result from the metal center. The present calculations show that the three low-lying electronic states possess linear configurations, in accordance with the evidences obtained in the previous studies (Douay and Bernath, 1990; Scurlock et al., 1994; Greetham and Ellis, 2000b). Accordingly, we scan the M-NC (M = Ca or Sr) PECs to examine the interfering states. Figure 1 shows the PECs of the lowest 6 electronic states for the CaNC molecule. The ground state and the first excited state of CaNC are the ^2^Σ^+^ and ^2^Π states, respectively. Higher electronic states include the B^2^Σ^+^, C^2^∆, D^2^Π, and E^2^Σ^+^ states. All states have smooth curves towards the dissociation limit. Based on the molecular orbital coefficients, the unpaired electron is mainly located on the s orbital of the Ca atom for the X^2^Σ^+^ state. And it transits to the p orbital of the Ca atom for the A^2^Π state (The relevant molecular orbitals are shown in Supplementary Figure S1). The second excited state is the B^2^Σ^+^ state lying ∼1800 cm^−1^ above the A^2^Π state, as shown in the inset panel of Figure 1. This energy separation indicates that the nearby B^2^Σ^+^ state should not come into play as an interference state. As a comparison, the energy gap of SrOH between its first and second excited states is ∼1700 cm^−1^ (Nakagawa et al., 1983), while the molecule has been experimentally cooled to the ultracold regime.

Between the A^2^Π and X^2^Σ^+^ states, the energy gap of CaNC is ∼16000 cm^−1^. After including the zero-point energy (ZPE) correction, the adiabatic excitation energy is calculated as 16218.3 cm^−1^ (see Table 1). We have also carried out calculations by scanning the Ca-N distance with all other degrees of freedom optimized. The corresponding adiabatic excitation energy is calculated as 16217.9 cm^−1^. The little difference between the two adiabatic excitation energies indicates that the N and C atoms move very little during the elongation of the Ca-N bond. Moreover, the calculated adiabatic excitation energy (16218 cm^−1^) is in excellent agreement with the experimental value of 16229.3 cm^−1^ (Scurlock et al., 1994). This energy gap is equivalent to ∼617 nm wavelength for the major laser pump. This reveals two additional features of the molecule. First, the excitation energy falls into the region of red color. Therefore, the incidence energy from laser pumps is not high and would not destabilize the molecule. In fact, as shown in Figure 1, the minimum of the A^2^Π state is well below the dissociation limit of the X^2^Σ^+^ state. Secondly, the ∼617 nm wavelength is easier to be accessible by laser gratings compared to its ultraviolet counterpart.

**TABLE 1:** Spectroscopic constants of the ground state and the first excited state of CaNC and SrNC.

State	Method	*T*_*e*_ (cm^−1^)^a^	*R*_*e*_ (Å)^b^	*ω*_*e*_ (cm^−1^)^c^	*ω*_*e*_ *χ*_*e*_ (cm^−1^)	*μ*_*e*_ (Debye)
CaNC						
X^2^Σ^+^	This work^d^		2.246	407.7	1.71	6.07
	This work^e^		2.246	404.4	1.71	6.07
	Calc		2.32^f^, 2.27^g^	392^h^, 330.6^i^		6.08^j^
	Exp		2.2065^g^	399^h^		6.84^g^
A^2^Π	This work^d^	16218.3	2.225	423.1	1.76	5.94
	This work^e^	16217.9	2.225	423.0	1.76	5.94
	Calc			344.7^i^		5.91^j^
	Exp	16229.3^g^				6.10^g^
SrNC						
X^2^Σ^+^	This work^d^		2.346	342.6	1.25	6.23
	This work^e^		2.345	342.9	1.26	6.27
	Calc			318^h^		
	Exp			338^h^		
A^2^Π	This work^d^	15051.0	2.312	363.0	1.23	5.48
	This work^e^	15047.6	2.312	363.2	1.23	5.49

a *T*_*e*_ is the adiabatic excitation energy referred to the ground state after the ZPE correction.

b *R*_*e*_ is the equilibrium bond length of the M-N bond.

c The harmonic vibrational constant (*ω*_*e*_) of the M-NC stretching mode.

d Results from PECs obtained by fixing other parameters when scanning the M-N bond lengths.

e Results from PECs obtained by optimizing other parameters when scanning the M-N bond lengths.

f Ref. (Steimle et al., 1992).

g Ref. (Scurlock et al., 1994).

h Ref. (Lanzisera and Andrews, 1997).

i Ref. (Nanbu et al., 1997).

j Ref. (Isaev and Berger, 2016).

It is worthy to add that the minimum configurations of all calculated states are between 2.0 Å and 2.3 Å. Especially, the minimum configurations of A^2^Π and X^2^Σ^+^ states are very close. The calculated equilibrium Ca-N bond length is 2.246 Å for the X^2^Σ^+^ state, and 2.225 Å for the A^2^Π state. Similar to the adiabatic excitation energy calculations, the relaxation or non-relaxation of other degrees of freedom during the PEC scans makes very little difference to the equilibrium Ca-N bond length. The close values of Ca-N bond lengths between A^2^Π and X^2^Σ^+^ states indicate that the molecular geometry does not have a significant change during the vibronic transition. Accordingly, a diagonal FCF matrix would be expected. This will be discussed in more details in the later sections.

**FIGURE 2:**
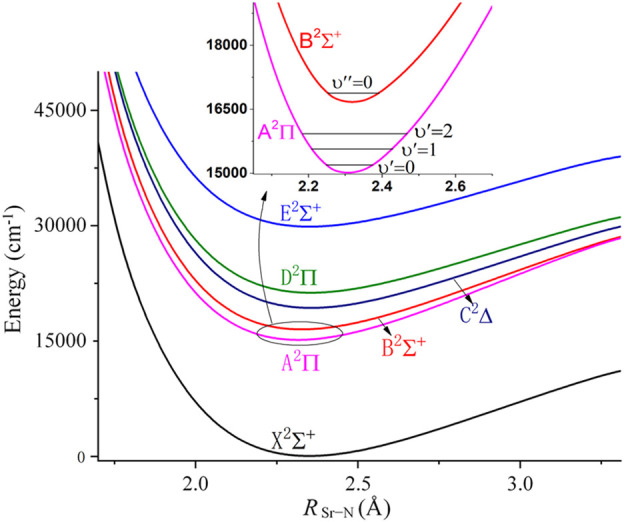
Potential energy curves of linear SrNC along R_Sr-N_, with R_N-C_ fixed at 1.183Å.

Figure 2 shows the PECs for the lowest 6 electronic states of the SrNC molecule. The electronic structure is similar to that of CaNC, where the ^2^Σ^+^ state is the ground state, and higher electronic states are the A^2^Π, B^2^Σ^+^, C^2^∆, D^2^Π and E^2^Σ^+^ states, respectively. The unpaired electron locates on the s orbital of the Sr atom for the X^2^Σ^+^ state, and on the p orbital of the Sr atom for the A^2^Π state. The second excited state is also a ^2^Σ^+^ state and it is ∼1700 cm^−1^ above the A^2^Π state (shown in the inset panel of Figure 2). This energy gap is comparable to that of SrOH as mentioned above. The energy separation between A^2^Π and X^2^Σ^+^ state is ∼15000 cm^−1^. After including the ZPE correction, the adiabatic excitation energy is calculated as 15051.0 cm^−1^ (based on PECs obtained by fixing other degrees of freedom), which is equivalent to ∼664 nm wavelength for the major laser pump. The adiabatic excitation energy which is based on PECs obtained by optimizing other degrees of freedom is calculated as 15047.6 cm^−1^. On the other hand, the calculated adiabatic excitation energy of SrNC is lower than that of CaNC. This may be understood as resulting from the smaller HOMO-LUMO energy gap for the Sr atom than that for the Ca atom.

**FIGURE 3:**
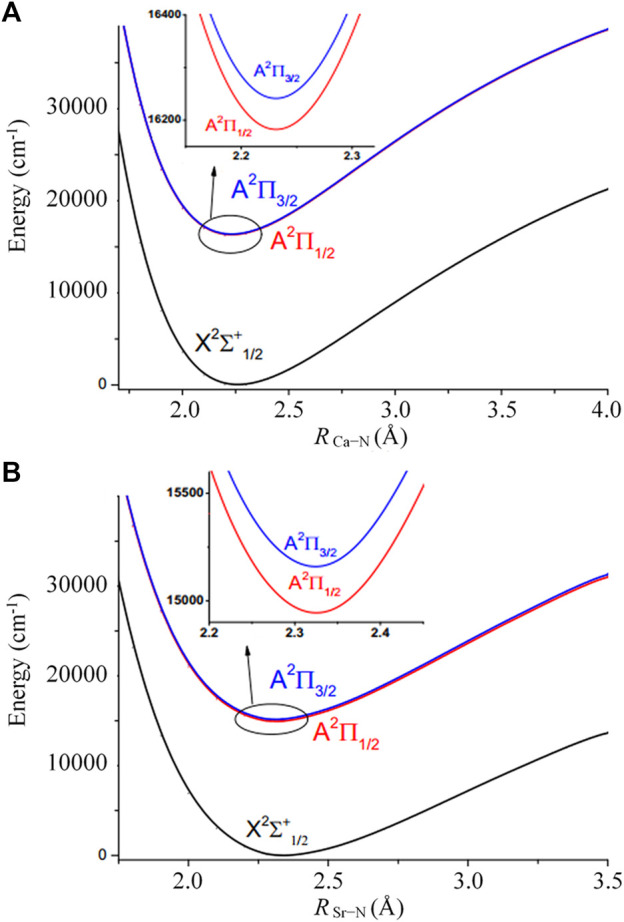
Potential energy curves for the X^2^Σ_1/2_^+^, A^2^Π_1/2_ and A^2^Π_3/2_ states of **(A)** CaNC and **(B)** SrNC obtained by fixing other parameters when scanning the M-N bond lengths.

It should not be ignored that the spin-orbit coupling (SOC) effect may play an important role, especially for molecules involving heavy elements (Fu et al., 2016a; Fu et al., 2016b; Fu et al., 2017; Xia et al., 2017). Figure 3A and Figure 3B show the PECs including SOC for CaNC and SrNC, respectively. It can be seen that the degenerate A^2^Π state splits after including the SOC effect. The energy gaps between the split states are ∼68 cm^−1^ and ∼282 cm^−1^ for CaNC and SrNC, respectively. The adiabatic excitation energy between the ground and the lowest excited state for CaNC is calculated as 16188.1 cm^−1^, which excellently agrees with the experimental value of 16190.3 cm^−1^ (Steimle et al., 1992). The excitation energy between the X^2^Σ_1/2_^+^ and A^2^Π_3/2_ states also excellently agrees with the experimental value.

For the SrNC molecule, on the other hand, the adiabatic excitation energy is calculated as 14914.8 cm^−1^, in excellent agreement with the experimental value of 14903.7 cm^−1^ (Douay and Bernath, 1990). The adiabatic excitation energy between the X^2^Σ_1/2_^+^ and A^2^Π_3/2_ states also very well agrees with the experimental value. The SOC effect is more evident than CaNC in terms of both the energy split of the A^2^Π state, and the energy shift of the adiabatic excitation energy.

### 3.2 Spectroscopic constants

Table 1 shows the adiabatic excitation energy (including the ZPE correction), equilibrium M-N bond length (M = Ca, Sr), M-NC harmonic stretching frequency, anharmonic term, and the PDM for CaNC and SrNC. The previous experimental and theoretical values are also listed for comparison.

For the CaNC molecule, the calculated adiabatic excitation energy excellently agrees with the experimental value. This indicates that the selected computation levels of theory and basis sets appropriately determine the geometry equilibria and the electronic states. More importantly, these give an accurate estimation of the laser wavelengths used in the cooling cycles (see the later section for more discussion). In addition, the PDM is calculated as ∼6 Debye, which is in very good agreement with the experimental measurement (∼6.8 Debye). This ensures the quality of the wavefunction. Noticeably, the large electric dipole moment (∼6 Debye) implies the Ca-N bond has a strong ionic bond character, rendering this molecule attractive for laser cooling applications (Isaev and Berger, 2016). As an interesting comparison, the CaCCH molecule (Xia et al., 2021), which is isoelectronic to CaNC, has an electric dipole moment only ∼3 Debye. In addition, the Ca-N bond length in CaNC is calculated as 2.246 Å, shorter than the Ca-C bond length in CaCCH (2.361 Å). These imply that the Ca-NC interaction is more ionic than the Ca-CCH interaction.

In terms of geometry and spectroscopic constants, the equilibrium Ca-N bond length of the X^2^Σ^+^ state agrees well with the experimental measurement. The corresponding bond length for the A^2^Π state is calculated as 2.225 Å. The harmonic stretching frequency of the Ca-NC mode is calculated as 404.4 cm^−1^, in very good agreement with experimental value (399 cm^−1^) (Lanzisera and Andrews, 1997). The anharmonic term is found to be very small. These indicate that the matrix effect to the vibrations is also small. Importantly, the FCF calculation relies on the geometry constants as well as harmonic frequencies. Therefore, the very good agreement of these constants with experimental measurements provides a reliable basis for FCF calculations.

For the SrNC molecule, less experimental data is available. The calculated stretching frequency of the Sr-NC mode is in very good agreement with experimental values. Unfortunately, no experimental data is available in terms of the geometry constants. Yet given the very good agreement achieved for spectroscopic constants of CaNC, and the very good agreement of Sr-NC stretching frequency, the corresponding calculated results should be an accurate estimation for the geometry constants. Similar to the CaNC molecule, the anharmonic term is found very small for the Sr-NC stretching.

The PDM of SrNC at its ground electronic state is calculated as 6.23 Debye. This is slightly larger than the PDM of CaNC at its ground state. For the first excited state, the PDMs for both molecules decrease, together with a shrink of the metal-N bond lengths. These might suggest that metal-NC interaction becomes less ionic when the electron is promoted to the higher orbital. The similar observation can also be found for the molecule pair of CaCCH and SrCCH (Xia et al., 2021).

Table 2 shows the spectroscopic constants of the CaNC and SrNC molecules with the SOC correction included. For the CaNC molecule, the SOC constant is calculated as 68.3 cm^−1^, in very good agreement with the experimental value. For the SrNC molecule, as expected, the SOC constant increases to 283.6 cm^−1^. The adiabatic excitation energy including SOC for CaNC is red-shifted by 31.1 cm^−1^, and the adiabatic excitation energy including SOC for SrNC is red-shifted by 135.0 cm^−1^.

**TABLE 2:** The calculated spectroscopic constants of several Ω states of CaNC and SrNC.

State	Method	*T*_*e*_ (cm^−1^)^a^	*R*_*e*_ (Å)^b^	*ω*_*e*_ (cm^−1^)^c^	*ω*_*e*_ *χ*_*e*_ (cm^−1^)	*A* (cm^-1^)^d^
CaNC						
X^2^Σ^+^_1/2_	This work^e^		2.246	404.5	1.71	
	This work^f^		2.246	404.4	1.71	
A^2^Π_1/2_	This work^e^	16188.1	2.225	423.1	1.78	
	This work^f^	16186.8	2.225	423.0	1.77	
	Exp	16190.3^g^				
A^2^Π_3/2_	This work^e^	16256.2	2.225	423.1	1.72	68.1
	This work^f^	16255.2	2.225	423.0	1.72	68.3
	Exp	16267.9^g^				77.7^g^
SrNC						
X^2^Σ^+^_1/2_	This work^e^		2.346	342.9	1.25	
	This work^f^		2.345	342.6	1.26	
A^2^Π_1/2_	This work^e^	14914.8	2.312	363.0	1.22	
	This work^f^	14912.6	2.313	362.8	1.22	
	Exp	14903.7^h^				
A^2^Π_3/2_	This work^e^	15196.9	2.311	363.5	1.36	282.0
	This work^f^	15196.2	2.312	363.3	1.36	283.6
	Exp	15205.1^h^				301^h^

a Te is the adiabatic excitation energy referred to the ground state after the ZPE correction.

b Re is the equilibrium bond length of the M-N bond.

c The harmonic vibrational constant (ω_e_) of the M-NC stretching mode.

d A is the spin-orbital coupling constant.

e Results from PECs obtained by fixing other parameters when scanning the M-N bond lengths.

f Results from PECs obtained by optimizing other parameters when scanning the M-N bond lengths.

g Ref. (Steimle et al., 1992)

h Ref. (Douay and Bernath, 1990)

It should be stressed at the end of this sub-section that the CaNC and SrNC stand out from other ultracold polyatomic molecular candidates in terms of their significantly strong molecular polarity. To the best of our knowledge, they have larger PDM (∼6 Debye) than other ultracold polyatomic molecular candidates currently known. For comparisons, the PDM for CaF (Childs et al., 1984) and CaCCH (Xia et al., 2021) are ∼3 Debye. More comparisons can be found in the supplementary material (Supplementary Table S1). Such a large dipole moment would facilitate the trapping of cold molecules for further applications (Grimm et al., 2000), because the molecules would respond more sensitively to the external electric field. In addition, the dipole-dipole interaction between the MNC (M = Ca or Sr) molecules should be more evident than their peers. Comparing to the atom-atom interaction, on the other hand, the dipole-dipole interaction is a long-range van der Waals interaction. Previous studies have shown that van der Waals interactions play important roles in molecular reaction dynamics (Skouteris et al., 1999; Shen et al., 2017; Cao et al., 2019; Wu et al., 2019). Hence, the long-range dipole-dipole interaction might lead to discoveries of new quantum effects, based on which novel technologies could be developed (Chen and Yan, 2019).

### 3.3 Laser cooling schemes

It requires several conditions for a molecule to be favored for direct laser cooling: a diagonal FCF matrix, a short lifetime for the excited state, no interference from intermediate electronic states, and no state-crossing nearby the state of interests. From the above discussions, we have shown that there are no intermediate electronic states interfering. In addition, the geometry and spectroscopic constants suggest a diagonal FCF matrix. Table 3 shows the FCFs and VBRs for the A^2^Π (000) → X^2^Σ^+^ (υ_1_υ_2_υ_3_) transitions, where υ_1_, υ_2_, and υ_3_ stand for the M-NC stretching mode, bending mode, and the MN-C stretching mode, respectively (M = Ca or Sr). The (000) represents the ground vibrational state. The FCF for the main transition (A^2^Π (000) → X^2^Σ^+^ (000)) is calculated as 0.9101 for the CaNC molecule. The corresponding FCF for SrNC is calculated as 0.9528. For both molecules, the FCFs (A^2^Π (000) → X^2^Σ^+^ (*υ*_1_ *υ*_2_ *υ*_3_)) show a rapid decay pattern, which is desirable for designing laser cooling cycles. The VBRs show very similar patterns to those of FCFs. The accumulated FCFs for CaNC and SrNC are 0.99991 and 0.99994, respectively by utilizing 6 and 4 laser pumps. These indicate that nearly 10000 photons can be scattered for each molecule, and thus the molecules can be cooled to the ultracold regime. It is worth mentioning that the vibronic effects will alter the FCFs to some extent and would have to be calculated to accurately give FCFs (for example, to the level of 10^−5^ or so) (Paul et al., 2019; Zhang et al., 2021).

**TABLE 3:** The calculated Franck-Condon factors (FCFs) and vibrational branching ratios (VBRs) of the A^2^Π (000) → X^2^Σ^+^ (*υ*_1_ *υ*_2_ *υ*_3_) transitions for CaNC and SrNC.

A^2^Π (000) → X^2^Σ^+^ (*υ*_1_ *υ*_2_ *υ*_3_)	FCF	Accumulated FCF	VBR
CaNC			
A^2^Π (000) → X^2^Σ^+^ (000)	9.1007 × 10^−1^	9.1007 × 10^−1^	9.0382 × 10^−1^
A^2^Π (000)→ X^2^Σ^+^ (100)	7.1171 × 10^−2^	9.8124 × 10^−1^	7.6402 × 10^−2^
A^2^Π (000)→ X^2^Σ^+^ (020)	1.5417 × 10^−2^	9.9666 × 10^−1^	1.6072 × 10^−2^
A^2^Π (000)→ X^2^Σ^+^ (200)	1.6506 × 10^−3^	9.9831 × 10^−1^	1.9193 × 10^−3^
A^2^Π (000)→ X^2^Σ^+^ (120)	1.2057 × 10^−3^	9.9952 × 10^−1^	1.3603 × 10^−3^
A^2^Π (000) → X^2^Σ^+^ (040)	3.9176 × 10^−4^	9.9991 × 10^−1^	4.2902 × 10^−4^
SrNC			
A^2^Π (000) → X^2^Σ^+^ (000)	9.5279 × 10^−1^	9.5279 × 10^−1^	9.4940 × 10^−1^
A^2^Π (000) → X^2^Σ^+^ (100)	4.6592 × 10^−2^	9.9938 × 10^−1^	4.9913 × 10^−2^
A^2^Π (000)→ X^2^Σ^+^ (200)	4.5006 × 10^−4^	9.9983 × 10^−1^	5.1927 × 10^−4^
A^2^Π (000) → X^2^Σ^+^ (001)	1.0996 × 10^−4^	9.9994 × 10^−1^	1.7165 × 10^−4^

The radiative lifetime *τ* should be short in favor of vertical transitions. The corresponding lifetimes for CaNC and SrNC are calculated as 15.5 and 15.8 ns, respectively. As a reference, the YbF molecule has been experimentally cooled to 100 μK and its radiative lifetime was measured as 28 ns (Lim et al., 2018). Obviously, the short radiative lifetimes meet the requirement for selecting laser-cooling candidates.

In addition to questing the feasibility of direct laser cooling, it is also interesting to know the temperature limit towards which molecules can be cooled. The Doppler temperature is evaluated accordingly. For the CaNC and SrNC molecules, the Doppler temperatures are evaluated as 246.6 and 242.0 µK, respectively. As a comparison, the Doppler temperatures for the isoelectronic molecules CaCCH and SrCCH are 153.5 and 141.1 µK, respectively. Another quantity usually used to characterize the temperature limit by laser cooling is the recoil temperature. The recoil temperatures for CaNC and SrNC are estimated as 762.0 and 381.2 nK, respectively, while for CaCCH and SrCCH the recoil temperatures are 706.5 and 337.7 nK, respectively.

**FIGURE 4:**
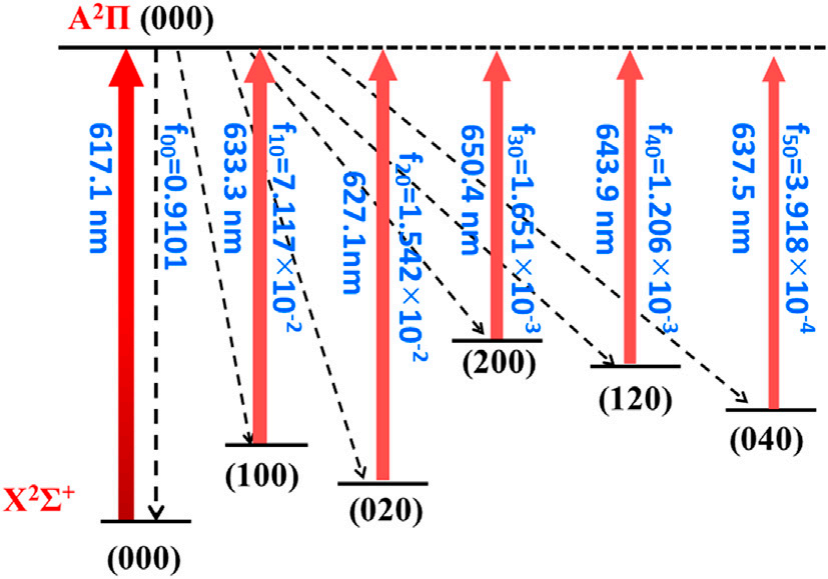
The constructed cooling scheme for CaNC using the A^2^Π (000) → X^2^Σ^+^ (*υ*_1_ *υ*_2_ *υ*_3_) transitions. The arrow colors qualitatively represent the laser wavelengths by different levels of transparency. The rotational branching can be eliminated by driving the *J* = 1 → *J'* = 0 transition.

**FIGURE 5:**
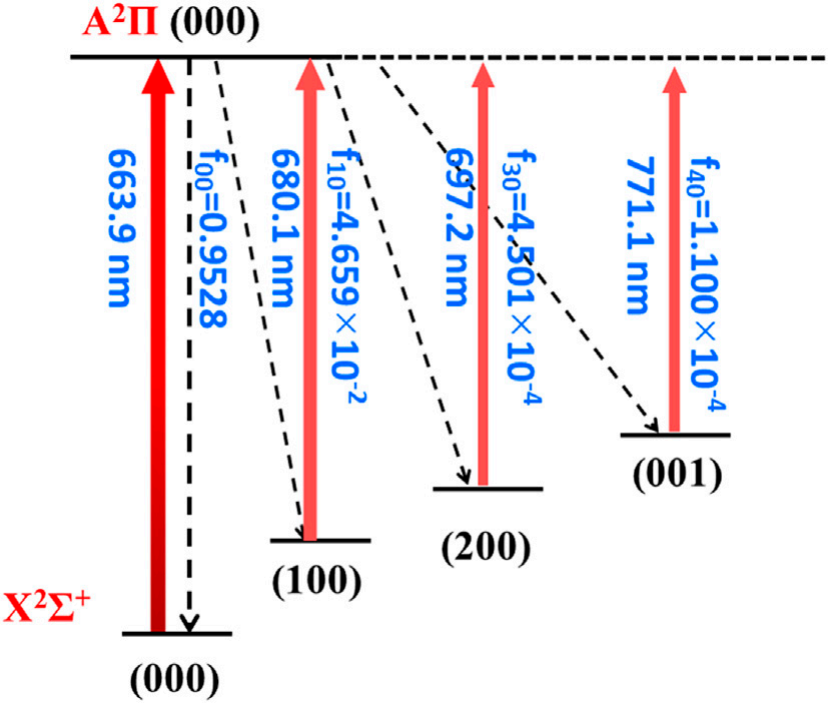
The constructed cooling scheme for SrNC using the A^2^Π (000) → X^2^Σ^+^ (*υ*_1_ *υ*_2_ *υ*_3_) transitions. The arrow colors qualitatively represent the laser wavelengths by different levels of transparency. The rotational branching can be eliminated by driving the *J* = 1 → *J'* = 0 transition.

Overall, the laser cooling schemes are constructed and shown in Figure 4 and Figure 5. The transitions are between X^2^Σ^+^ (*υ*_1_ *υ*_2_ *υ*_3_) and A^2^Π (000) states. Given the excellent agreement between experimental and calculated values in terms of adiabatic excitation energies and vibrational frequencies, the wavelengths for the laser pumps can be accurately determined. The wavelengths for laser pumps range from 617.1 to 650.4 nm for CaNC, and from 663.9 to 771.1 nm for SrNC. The main transition is between the X^2^Σ^+^ (000) and A^2^Π (000) states, so that the excited molecules statistically prefer returning to the X^2^Σ^+^ (000) state after scattering photons. The repump lasers re-excite molecules back to the A^2^Π (000) state to reduce the leakage to other vibrational states. The transition arrows shown in Figure 4 and Figure 5 are qualitatively represented by red colors with different levels of transparency, as the wavelengths of laser pumps fall in the region from red to near-infrared. The energy levels are qualitatively placed. The FCFs are shown with three decimals for better readability.

## 4 Conclusion

In this work, we establish two polyatomic candidates, CaNC and SrNC, which are suitable for laser cooling to the ultracold regime. These molecules possess impressively large electric dipole moments, which are preferred for applications using an external electric field. High-level *ab initio* calculations are carried out to reveal electronic structures of these molecules, and the calculated spectroscopic constants are in very good agreement with the available experimental data. For each molecule, the obtained Franck-Condon factor matrix is highly diagonal. The estimated radiative lifetimes for CaNC and SrNC are 15.5 and 15.8 ns, respectively, which are short enough for rapid and efficient laser cooling. By choosing suitable spin-orbit states, we construct feasible laser cooling schemes for CaNC and SrNC, each of which allows scattering nearly 10000 photons for direct laser cooling. These calculated results indicate that CaNC and SrNC are excellent ultracold polyatomic candidates with strong polarity. We hope the present work could stimulate experimental research interests in these two promising molecules.

## Data Availability

The original contributions presented in the study are included in the article/Supplementary Material, further inquiries can be directed to the corresponding authors.
