# Supplementary material for: Production of ultracold polyatomic molecules with strong polarity by laser cooling: A detailed theoretical study on CaNC and SrNC
Source: Front Chem. 2022 Sep 23;10:1009986. doi: 10.3389/fchem.2022.1009986 (PMC9538186; doi:10.3389/fchem.2022.1009986)
Supplement: Supplementary file 1 [file DataSheet1.pdf]

Supplementary material for:

Production of ultracold polyatomic molecules with strong polarity by laser cooling: A detailed theoretical study on CaNC and SrNC

Wensha Xia[1,2] †, Jianwei Cao[1] †, Qing Lu[1], Wensheng Bian[1,2]**

1. Beijing National Laboratory for Molecular Sciences, Institute of Chemistry, Chinese Academy of Sciences, 100190, Beijing, China

2. School of Chemical Sciences, University of Chinese Academy of Sciences, Beijing 100049, China

Table of Contents

Table S1. Comparison of ground state permanent dipole moment of molecules for direct laser cooling.

Figure S1. Relevant molecular orbitals of CaNC for the ground and first excited states (isovalue 0.02).

Table S1. Comparison of ground state permanent dipole moment of molecules for direct laser cooling.

Molecule candidate	Permanent dipole moment (Debye)	Reference
CaNC	6.1	This work
SrNC	6.2	This work
CaF	3.1	(1)
SrF	1.4	(2)
CaOH	1.5	(3)
SrOH	1.9	(3)
CaCCH	3.9	(4)
CaCCYb	1.0	(5)
CaCCAl	4.4	(5)
$CaOCH_3$	1.6	(6)

There are other molecule candidates with different alkaline earth metal centers, (i.e. Be, Mg, Ba, Ra), but they are not listed in the table. It is not expected that their permanent dipole moment would be significantly different from the Ca or Sr counterpart.

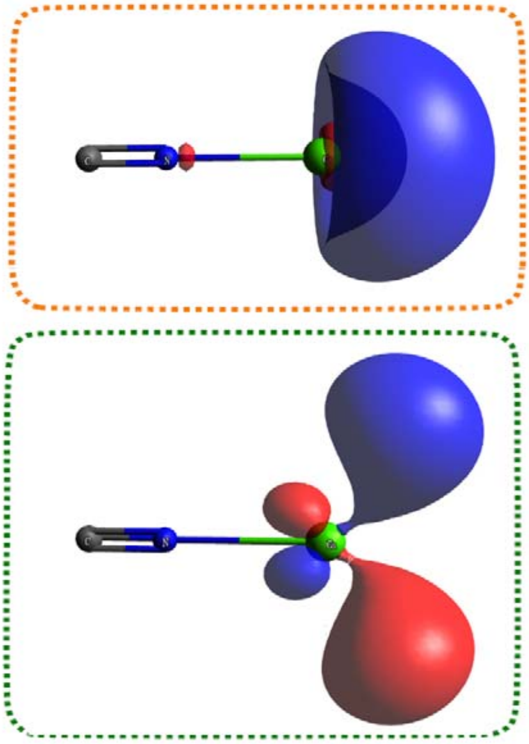

Figure S1. Relevant molecular orbitals of CaNC for the ground and first excited states (isovalue 0.02). Top: Highest occupied molecular orbitals (HOMO) of CaNC; Bottom: Lowest unoccupied molecular orbitals (LUMO) of CaNC.

References:

1. Childs WJ, Goodman LS, Nielsen U, Pfeufer V. Electric - dipole moment of CaF (X $^2\Sigma^+$) by molecular beam, laser - rf, double - resonance study of Stark splittings. The Journal of Chemical Physics. 1984;80(6):2283-7.
2. Ernst WE, Kändler J, Kindt S, Törring T. Electric dipole moment of SrF X $^2\Sigma^+$ from high-precision stark effect measurements. Chemical Physics Letters. 1985;113(4):351-4.
3. Steimle TC, Fletcher DA, Jung KY, Scurlock CT. Molecular beam optical Stark spectroscopy of calcium monocyanide. The Journal of Chemical Physics. 1992;97(5):2909-19.
4. Xia W, Ma H, Bian W. Production of ultracold CaCCH and SrCCH molecules by direct laser cooling: A theoretical study based on accurate ab initio calculations. The Journal of Chemical Physics. 2021;155:204304.
5. O'Rourke MJ, Hutzler NR. Hypermetallic polar molecules for precision measurements. Physical Review A. 2019;100(2):022502.
6. Namiki K-iC, Robinson JS, Steimle TC. A spectroscopic study of $CaOCH_3$ using the pump/probe microwave and the molecular beam/optical Stark techniques. The Journal of Chemical Physics. 1998;109(13):5283-9.
